# Publisher Correction: A transcriptomic axis predicts state modulation of cortical interneurons

**DOI:** 10.1038/s41586-022-05209-8

**Published:** 2022-08-25

**Authors:** Stéphane Bugeon, Joshua Duffield, Mario Dipoppa, Anne Ritoux, Isabelle Prankerd, Dimitris Nicoloutsopoulos, David Orme, Maxwell Shinn, Han Peng, Hamish Forrest, Aiste Viduolyte, Charu Bai Reddy, Yoh Isogai, Matteo Carandini, Kenneth D. Harris

**Affiliations:** 1 UCL Queen Square Institute of Neurology, University College London, London, UK; 2 Center for Theoretical Neuroscience, Columbia University, New York, NY USA; 3 Department of Physics, University of Oxford, Oxford, UK; 4 UCL Institute of Ophthalmology, University College London, London, UK; 5 Sainsbury Wellcome Centre for Neural Circuits and Behaviour, University College London, London, UK

**Keywords:** Neural circuits, Striate cortex

Correction to: *Nature* 10.1038/s41586-022-04915-7Published online 6 July 2022

In the version of this article initially published, the wrong version of Fig. 6 was presented, specifically in the far-right panel of Fig. 6a, where the *x*-axis units now run “5, 10, 20, 40,” rather than “2, 2.5, 3, 3.5” as originally published. In the same axis, the legend now reads “Time constant (ms)” rather than “Time constant (log τ) (log(ms))” as originally published. The changes have been made to the HTML and PDF versions of the article.

