# Supplementary material for: A transcriptomic axis predicts state modulation of cortical interneurons
Source: Nature. 2022 Jul 6;607(7918):330–8. doi: 10.1038/s41586-022-04915-7 (PMC9279161; doi:10.1038/s41586-022-04915-7)
Supplement: Supplementary file 1 — This file contains Supplementary Discussion; legends for Supplementary Data 1–6 and Supplementary References. [file 41586_2022_4915_MOESM1_ESM.pdf]

nature portfolio

https://doi.org/10.1038/s41586-022-04915-7

Supplementary information

A transcriptomic axis predicts state modulation of cortical interneurons

In the format provided by the authors and unedited

A transcriptomic axis predicts state modulation of cortical interneurons

Stephane Bugeon[1*], Joshua Duffield[1], Mario Dipoppa[1,2], Anne Ritoux[1], Isabelle Prankerd[1], Dimitris Nicoloutsopoulos[1], David Orme[1], Maxwell Shinn[1], Han Peng[3], Hamish Forrest[1], Aiste Viduolyte[1], Charu Bai Reddy[1,5], Yoh Isogai[4], Matteo Carandini[5], Kenneth D. Harris[1*]

[1]UCL Queen Square Institute of Neurology, University College London, London, UK. [2]Columbia University Center for Theoretical Neuroscience, New York NY, USA. [3]Department of Physics, University of Oxford, Oxford, UK. [4]UCL Sainsbury Wellcome Centre, University College London, London, UK. [5]UCL Institute of Ophthalmology, University College London, London, UK. *Correspondence: s.bugeon@ucl.ac.uk, kenneth.harris@ucl.ac.uk.

Table of contents

Supplementary Discussion 2

Supplementary Data File Legends 3

Supplementary Discussion

The diversity that we observed across Subtypes may explain why previous reports of state modulation of different interneuron types, based on transgenic lines, have at times given apparently conflicting results. Previous work has uniformly shown that the activity of Vip-Cre labelled neurons in V1 is enhanced by running[1–5] and our data are fully consistent with this. Recent work has shown that most V1 cells labelled in Ndnf-Cre mice fire more in aroused states[6]. This is again consistent with our results: Ndnf is found in most Lamp5 Subtypes but not Lamp5-Lsp1, which was the only Lamp5 Subtype with significantly negative state modulation. Measurements of running modulation in Pvalb-Cre mice (which will label mainly basket cells) have shown mixed results[1,2,4], which may be explained by an effect of cell depth on running modulation: running primarily suppressed Pvalb cells above 300 μm and primarily suppresses Pvalb cells below that depth[1]. Our data involved only cells above 300 μm depth and showed close to uniform negative state modulation in Pvalb-Tac1 (putative basket) cells but positive modulation in Pvalb-Vipr2 (putative chandelier), consistent with data from Vipr2-Cre mice[7]. We speculate that the deeper-layer cells positively modulated in Pvalb-Cre mice correspond to additional Pvalb-Tac1 Subtypes not recorded here. An additional factor that may explain differing results in previous work is light level. Work in Sst-Cre mice has shown that running suppresses activity in pitch darkness, but has mixed effects in light[1,2,4,6,8]; our data were conducted in light, and we speculate that the apparent mixed effects seen in Sst-Cre mice might reflect a Type difference, with running suppressing Sst-Tac1 cells while activating Sst-Reln cells.

Cell-type-specific cholinergic receptor expression may contribute mechanistically to the different *in vivo* state modulation of different inhibitory types, but is unlikely to be the only mechanism doing so. Acetylcholine levels are largest in locomotion and lowest in synchronized states, and state modulation of at least some interneuron classes depends on cell-type-specific nicotinic and muscarinic currents[2,9]. However direct cholinergic input is unlikely to be the only factor mediating state dependence of an interneuron class: interneurons receive input from pyramidal cells, and from each other in specific ways such as the well-known "disinhibitory circuit"[2,9,10]. Nevertheless, the correlation of cholinergic receptor expression and state modulation we observed suggests that cell-type-specific cholinergic modulation might play a substantial role, at least in superficial V1. In contrast, the fact that sensory tuning only showed major differences between Subclasses, and not finer Types and Subtypes, might reflect the fact the sensory tuning of inhibitory cells depends only on the pattern and tuning of their excitatory and inhibitory inputs. Determining the causal contributions of neuromodulatory receptors, excitatory and inhibitory inputs, and cellular physiology in generating different Subtypes' *in vivo* firing remains a topic for future research.

Although we have focused here on one dimension of arousal/desynchronization, the space of cortical states is unlikely to be one dimensional, as ongoing activity correlates with multiple dimensions of V1 excitatory cell activity[11]. Characterizing this multidimensional space of cortical states and relating it to the diversity of inhibitory types remains an important topic for future work, as does understanding whether this relationship varies between different cortical regions, and whether behavioural tasks or other classes of sensory stimuli could reveal further distinctions between inhibitory cell types. High-throughput application of the current methods will make this possible.

1. Dipoppa, M. et al. Vision and Locomotion Shape the Interactions between Neuron Types in Mouse Visual Cortex. Neuron 98, 602-615.e8 (2018).
2. Fu, Y. *et al.* A cortical circuit for gain control by behavioral state. *Cell* **156**, 1139–52 (2014).
3. Millman, D. J. *et al.* VIP interneurons in mouse primary visual cortex selectively enhance responses to weak but specific stimuli. *eLife* **9**, e55130 (2020).
4. Pakan, J. M. *et al.* Behavioral-state modulation of inhibition is context-dependent and cell type specific in mouse visual cortex. *eLife* **5**, e14985 (2016).
5. Reimer, J. *et al.* Pupil fluctuations track fast switching of cortical states during quiet wakefulness. *Neuron* **84**, 355–62 (2014).
6. Cohen-Kashi Malina, K. *et al.* NDNF interneurons in layer 1 gain-modulate whole cortical columns according to an animal's behavioral state. *Neuron* **109**, 2150-2164.e5 (2021).
7. Schneider-Mizell, C. M. *et al.* Structure and function of axo-axonic inhibition. *Elife* **10**, e73783 (2021).
8. Polack, P. O., Friedman, J. & Golshani, P. Cellular mechanisms of brain state-dependent gain modulation in visual cortex. *Nature neuroscience* (2013) doi:10.1038/nn.3464.
9. Muñoz, W., Tremblay, R., Levenstein, D. & Rudy, B. Layer-specific modulation of neocortical dendritic inhibition during active wakefulness. *Science* **355**, 954–959 (2017).
10. Lee, S., Kruglikov, I., Huang, Z. J., Fishell, G. & Rudy, B. A disinhibitory circuit mediates motor integration in the somatosensory cortex. *Nature neuroscience* **16**, 1662–70 (2013).
11. Stringer, C. *et al.* Spontaneous behaviors drive multidimensional, brainwide activity. *Science* **364**, 255 (2019).

Supplementary Data File Legends

Supplementary Data File 1 (.xlsx). Percentage of interneurons assigned to a Subtype. Number of interneurons recorded per session and per animal, and percentage of interneurons that were assigned to a Subtype at the end of the experimental pipeline (registration to *ex-vivo* slices, production of RCPs, spot calling and cell calling). In total, about 44% of recorded interneurons were characterized transcriptomically (most of the cells that could not be assigned were lost at the registration stage).

Supplementary Data File 2 (.xlsx). Padlock probe sequences. Name and sequence of the 556 padlock probes (73 to 80nt) targeting the cDNA sequences produced by reverse transcription. Each probe contains the same 20nt anchor sequence, a 20nt gene specific DNA barcode, and two arms complementary to the cDNA sequence.

Supplementary Data File 3 (.xlsx). Primer sequences. Name and sequence of the 556 primers used for reverse transcription of the mRNAs.

Supplementary Data File 4 (.xlsx). Dye probe sequences. Name and sequence of the 7 dye probes used for combinatorial imaging. Each 20nt DNA oligo was conjugated to a given dye. All dyes were conjugated at the 5′ end only, except from dp0 and dp6 which were conjugated at both ends.

Supplementary Data File 5 (.xlsx). Bridge probe sequences. Name and sequence of the 511 bridge probes used for combinatorial imaging, 1 for each gene and imaging round.

Supplementary Data File 6 (.xlsx). Reed-Solomon codes. Dye code for each gene, consisting of 7 numbers between 0 and 6, listing the dye probe that gene is assigned on each round.
