# Supplementary material for: A transcriptomic axis predicts state modulation of cortical interneurons
Source: Nature. 2022 Jul 6;607(7918):330–8. doi: 10.1038/s41586-022-04915-7 (PMC9279161; doi:10.1038/s41586-022-04915-7)
Supplement: Supplementary file 2 — Reporting Summary [file 41586_2022_4915_MOESM2_ESM.pdf]

nature portfolio

Corresponding author(s): Kenneth D Harris

Last updated by author(s): YYYY-MM-DD

Reporting Summary

Nature Portfolio wishes to improve the reproducibility of the work that we publish. This form provides structure for consistency and transparency in reporting. For further information on Nature Portfolio policies, see our Editorial Policies and the Editorial Policy Checklist.

Statistics

For all statistical analyses, confirm that the following items are present in the figure legend, table legend, main text, or Methods section.

n/a	Confirmed	
☐	☒	The exact sample size (*n*) for each experimental group/condition, given as a discrete number and unit of measurement
☐	☒	A statement on whether measurements were taken from distinct samples or whether the same sample was measured repeatedly
☐	☒	The statistical test(s) used AND whether they are one- or two-sided<br>*Only common tests should be described solely by name; describe more complex techniques in the Methods section.*
☐	☒	A description of all covariates tested
☐	☒	A description of any assumptions or corrections, such as tests of normality and adjustment for multiple comparisons
☐	☒	A full description of the statistical parameters including central tendency (e.g. means) or other basic estimates (e.g. regression coefficient) AND variation (e.g. standard deviation) or associated estimates of uncertainty (e.g. confidence intervals)
☐	☒	For null hypothesis testing, the test statistic (e.g. *F*, *t*, *r*) with confidence intervals, effect sizes, degrees of freedom and *P* value noted<br>*Give P values as exact values whenever suitable.*
☒	☐	For Bayesian analysis, information on the choice of priors and Markov chain Monte Carlo settings
☐	☒	For hierarchical and complex designs, identification of the appropriate level for tests and full reporting of outcomes
☐	☒	Estimates of effect sizes (e.g. Cohen's *d*, Pearson's *r*), indicating how they were calculated

Our web collection on statistics for biologists contains articles on many of the points above.

Software and code

Policy information about availability of computer code

Data collection	NIS-Elements (v5.20.02, build 1453, Nikon), ScanImage v4.2 (written in Matlab), Kilroy toolbox for Matlab (01/09/2019 version, https://github.com/ZhuangLab/storm-control; edits available at https://github.com/acycliq/storm-control)
Data analysis	The custom code written in Matlab (R2019b) to analyse and plot the processed data will be available upon reasonable request to the authors.<br>Suite2P toolbox for Matlab (01/05/2018 version, https://github.com/cortex-lab/Suite2P)<br>In situ data analysis code can be found at (16/07/2019 version, https://github.com/jduffield65/iss)<br>Registration code can be found at https://github.com/ha-ha-ha-han/NeuromicsCellDetection/, edits available at https://github.com/sbugeon/NeuromicsCellDetection, version 26/11/2021)<br>Facemap toolbox for Matlab (18/11/2018, https://github.com/MouseLand/facemap)<br>Intensify3D toolbox for Matlab (version 20/07/2020, https://github.com/nadavyayon/Intensify3D/blob/master/User_GUI_Intensify3D.m)

For manuscripts utilizing custom algorithms or software that are central to the research but not yet described in published literature, software must be made available to editors and reviewers. We strongly encourage code deposition in a community repository (e.g. GitHub). See the Nature Portfolio guidelines for submitting code & software for further information.

Data

Policy information about availability of data

All manuscripts must include a data availability statement. This statement should provide the following information, where applicable:

- Accession codes, unique identifiers, or web links for publicly available datasets
- A description of any restrictions on data availability
- For clinical datasets or third party data, please ensure that the statement adheres to our policy

Processed data (cellular calcium traces, gene detections, etc) are available at https://doi.org/10.6084/m9.figshare.19448531.v1.
The raw data (2-photon movies, transcriptomic images etc.) will be made available upon reasonable request.
Natural scenes were obtained from the ImageNet database (https://www.image-net.org/).

Field-specific reporting

Please select the one below that is the best fit for your research. If you are not sure, read the appropriate sections before making your selection.

☒ Life sciences ☐ Behavioural & social sciences ☐ Ecological, evolutionary & environmental sciences

For a reference copy of the document with all sections, see nature.com/documents/nr-reporting-summary-flat.pdf

Life sciences study design

All studies must disclose on these points even when the disclosure is negative.

Sample size	We did not use statistical methods to pre-determine sample sizes. However, our sample sizes are similar to those reported in previous publications using a similar approach (Xu et al., Science, 2020; Condylis et al., Science, 2022).
Data exclusions	Eight cells were excluded as they were assigned to a Subtype with less than 3 cells in total to avoid statistical analyses comparing groups with less than 3 observations. This exclusion criteria was not pre-established.
Replication	All experiments were performed on 4 independent animals, the results were reliably replicated across all animals.
Randomization	Our study did not contain experimental groups so randomization does not apply.
Blinding	Our study did not contain experimental groups so blinding does not apply.

Reporting for specific materials, systems and methods

We require information from authors about some types of materials, experimental systems and methods used in many studies. Here, indicate whether each material, system or method listed is relevant to your study. If you are not sure if a list item applies to your research, read the appropriate section before selecting a response.

Materials & experimental systems

n/a	Involved in the study	
☒	☐	Antibodies
☒	☐	Eukaryotic cell lines
☒	☐	Palaeontology and archaeology
☐	☒	Animals and other organisms
☒	☐	Human research participants
☒	☐	Clinical data
☒	☐	Dual use research of concern

Methods

n/a	Involved in the study	
☒	☐	ChIP-seq
☒	☐	Flow cytometry
☒	☐	MRI-based neuroimaging

Animals and other organisms

Policy information about studies involving animals; ARRIVE guidelines recommended for reporting animal research

Laboratory animals	Mice (Mus musculus) from 3 transgenic mouse lines: Gad2-T2a-NLS-mCherry (2 males and 2 females), Pvalb<tm1(cre)Arbr> (1 male), Sst<tm2.1(cre)Zjh> (2 males and 1 female), were used in this study. All mice were maintained on a C57BL/6 genetic background. Mice were aged between 12 and 15 weeks.
Wild animals	No wild animals involved.

Field-collected samples	No samples collected from field.
Ethics oversight	All experimental procedures were conducted in accordance with the UK Animals (Scientific Procedures Act) 1986. Experiments were performed at University College London under personal and project licences released by the Home Office following appropriate ethics review.

Note that full information on the approval of the study protocol must also be provided in the manuscript.
